# Collagen Content in Skin and Internal Organs of the Tight Skin Mouse: An Animal Model of Scleroderma

**DOI:** 10.1155/2013/436053

**Published:** 2013-10-24

**Authors:** Jayanthi Manne, Marina Markova, Linda D. Siracusa, Sergio A. Jimenez

**Affiliations:** ^1^Department of Microbiology and Immunology, Kimmel Cancer Center, Jefferson Medical College, Thomas Jefferson University, 233 South 10th Street, Philadelphia, PA 19107-5541, USA; ^2^Jefferson Institute of Molecular Medicine, Jefferson Medical College, Thomas Jefferson University, Suite 509 Bluemle Life Sciences Building, 233 South 10th Street, Philadelphia, PA 19107-5541, USA

## Abstract

The Tight Skin mouse is a genetically induced animal model of tissue fibrosis caused by a large in-frame mutation in the gene encoding fibrillin-1 (Fbn-1). We examined the influence of gender on the collagen content of tissues in C57BL/6J wild type (+/+) and mutant Tight Skin (*Tsk*/+) mice employing hydroxyproline assays. Tissue sections were stained with Masson's trichrome to identify collagen *in situ*. Adult *Tsk*/+ mice skin contains ~15% more collagen, on average, than skin from +/+ mice of the same gender. The heart of *Tsk*/+ males had significantly more collagen than that of +/+ males. No significant gender differences were found in lungs and kidney collagen content. Overall, the collagen content of *Tsk*/+ males and +/+ males was higher than that of their *Tsk*/+ and +/+ female counterparts, respectively. Our data confirm increased deposition of collagen in skin and hearts of *Tsk*/+ mice; however, the effects of the *Tsk* mutation on collagen content are both tissue specific and gender specific. These results indicate that comparative studies of collagen content between normal and *Tsk*/+ mice skin and internal organs must take into account gender differences caused by expression of the androgen receptor.

## 1. Introduction

Systemic sclerosis (SSc, scleroderma) is a systemic autoimmune disease of unknown etiology characterized by excessive accumulation of collagen in the skin and internal organs, including the gastrointestinal tract, lungs, heart, and kidneys [1]. Besides the often progressive fibrotic process, the pathogenesis of this disorder is characterized by severe microvascular alterations and distinct immunological abnormalities in cellular and humoral responses [2]. 

The etiologic factors involved in the development and progression of SSc are still unclear thus, study of animal models of the disease is likely to provide valuable information about its pathogenesis and allow identification of potentially effective therapeutic approaches. Mammalian model systems that reproduce all the features of human SSc are not available. Two spontaneous mouse mutations, Tight Skin (*Tsk*) and Tight Skin 2 (*Tsk2*), recapitulate some, but not all, features of this disease [3, 4]. The *Tsk* mutation occurred spontaneously in the inbred mouse strain B10.D2(58N)/Sn [5]. Homozygous *Tsk*/*Tsk* mice die *in utero* by day 8 of gestation, while heterozygous *Tsk*/+ mice survive to adulthood. *Tsk*/+ mice have thickened skin which is bound tightly to subcutaneous tissue under the dermis and lacks the pliability and elasticity of normal skin [5, 6]. Several other features in *Tsk*/+ mice include an enlarged skeleton and increased cartilage growth, emphysematous lungs, myocardial hypertrophy, and the presence of smaller tendons with tendon sheath hyperplasia [7–9]. The *Tsk* mutation is an autosomal dominant mutation on mouse chromosome 2 [5, 10]. Molecular analysis of *Tsk*/+ mice revealed a large, in-frame genomic duplication in the fibrillin-1 gene (*Fbn-1*) that includes exons 17–40 [11] and results in a mutant *Fbn-1* transcript that is 3 kb larger than the wild type *Fbn-1* transcript. 

In addition to *Tsk*, a second mouse mutant, called Tight skin 2 (*Tsk2*), was detected as a result of chemical mutagenesis [12]. The *Tsk2* mutation is distinct from the original *Tsk* mutation, since *Tsk2* maps to mouse chromosome 1 [13]. Heterozygous *Tsk2*/+ mice show an increased thickening of the dermis and excessive deposition of thick collagen fibers extending into the subdermal adipose tissue [14]. In addition to fibrosis, *Tsk2*/+ mice display infiltration of mononuclear cells in the lower dermis [14]. Both *Tsk* and *Tsk2* mice show similar histopathologic and biochemical abnormalities to those present in systemic sclerosis, and thus, these mutant mice serve as important models for this disease [4]. Their study has allowed the unraveling of novel mechanisms and pathways of relevance for the pathogenesis of this disease as well as for other fibrotic diseases [15–18]. 

Previous reports by several investigators have shown a 2-3-fold increase in collagen content in the skin of *Tsk*/+ mice compared to +/+ mice [6, 15–21]. However, most of these studies did not distinguish the gender or age of the mice. We report in this paper quantitative measurements of total collagen content in skin, lung, heart, and kidney samples from *Tsk*/+ mice compared to wildtype +/+ mice, with special emphasis on the importance of controlling for age, gender, and genetic background. Our results indicate that the large differences in collagen content between normal and *Tsk*/+ mice skin reported in some previous studies may reflect age and gender differences rather than the direct effect of the mutation. However, our data indeed confirm that there is increased deposition of collagen in skin and hearts of *Tsk*/+ mice.

## 2. Materials and Methods

### 2.1. Mice

C57BL/6J-pa +/+ *Tsk* mice were purchased from The Jackson Laboratory (Bar Harbor, ME, USA). The *Tsk* mutation was maintained on the C57BL/6J (B6) background by sequential backcrossing at Thomas Jefferson University as previously described [10, 11]. The B6 *Tsk*/+ and B6 +/+ mice used in this study were littermates at the N6 generation. All studies were reviewed and approved by the Institutional Animal Care and Use Committee.

### 2.2. Collection of Samples

A depilatory cream (Nair; Carter Wallace Inc., New York, NY, USA) was used to remove mouse hair. The skin along the dorsal side immediately below the neck and close to the tail region was removed completely. On both sides (left and right) of the dorsal midline, three regions were marked (a) anterior (interscapular region), (b) middle (midregion of the back), and (c) posterior (close to the tail). A total of 6 full thickness skin biopsies (4 mm in diameter) were excised from each region of the dorsum using sterile disposable dermal biopsy punches (Miltex, Inc., Bethpage, NY, USA). Whole heart, both lungs, and both kidneys were excised and included in this study.

### 2.3. Determination of Hydroxyproline Levels in Tissues

The full thickness skin biopsy samples including the panniculus carnosus and the fascia were separated from the subjacent muscle by dissection with a scalpel, and the resulting samples were acid-hydrolyzed in 6 N HCl (3 mL of 6 N HCl/skin punch). The heart, lung, or a single kidney was hydrolyzed in 9 mL of 6 N HCl. The samples were hydrolyzed overnight at 107°C and assayed for their hydroxyproline content as described [24]. The total content of hydroxyproline was determined from a standard curve prepared by dissolving hydroxyproline in distilled H_2_O and calculated based on the initial volume of each hydrolysate sample. The hydroxyproline content was expressed as *μ*g hydroxyproline per full thickness skin biopsy or per organ hydrolyzed. 

### 2.4. Histopathology of Tissues

Mice were euthanized by carbon dioxide inhalation, and skin, lungs, heart, and kidneys were collected. Nair depilatory cream was used to remove hair from the skin. Skin samples were then taken, pinned flat to dental wax, and fixed in 10% buffered formalin. Lungs were inflated through the trachea with 10% buffered formalin using a 5 mL syringe with a 27 G needle and then fixed in 10% buffered formalin. All tissues were embedded in paraffin and sectioned (5 micrometer thickness). Tissue sections were then stained with either hematoxylin and eosin or separately with Masson's trichrome.

### 2.5. Statistical Analysis

Statistical analysis of the 3 skin sites was carried out using a mixed effects 3-way analysis of variance, with sex, site, and phenotypes as fixed effects and mouse (nested within sex) as a random effect. This protocol was followed by a *t*-test (using standard errors derived from the above model) comparing individual means. A similar analysis was done separately for heart, lung, and kidney (left and right). 

## 3. Results

Our previous studies demonstrated that both age and gender can significantly influence collagen content in normal mouse tissues [25]. Therefore, we selected wildtype and *Tsk*/+ mutant adult mice within a narrow window of age (between 23 through 27 weeks after birth) for this study (Table 1). To detect differences attributable specifically to the *Tsk* mutation, we used genetically homogeneous mice of the C57BL/6J (B6) inbred strain [26] for all analyses. The *Tsk* mutation was backcrossed onto the B6 background for 6 generations, and +/+ littermates served as controls. Our study was composed of four groups: wildtype (+/+) females, wildtype males (+/+), mutant (*Tsk*/+) females, and mutant (*Tsk*/+) males. 

### 3.1. Hydroxyproline Content in the Skin Samples of C57BL/6J Wild Type +/+ and Mutant *Tsk*/+ Mice

 Total hydroxyproline content was assayed in skin biopsies obtained from the dorsal region of each group of mice. Three regions were marked on the dorsum: Site 1 was the interscapular region, Site 2 was the middle of the dorsum, and Site 3 was the posterior region, close to the tail. Skin biopsy punches were taken from both sides of the dorsal midline to ensure reliability in sample measurements. Since the amount of hydroxyproline did not vary between the left and right skin punches from the same site, the two values for each site were combined and the average was subjected to statistical analysis. 

**Figure 1:**
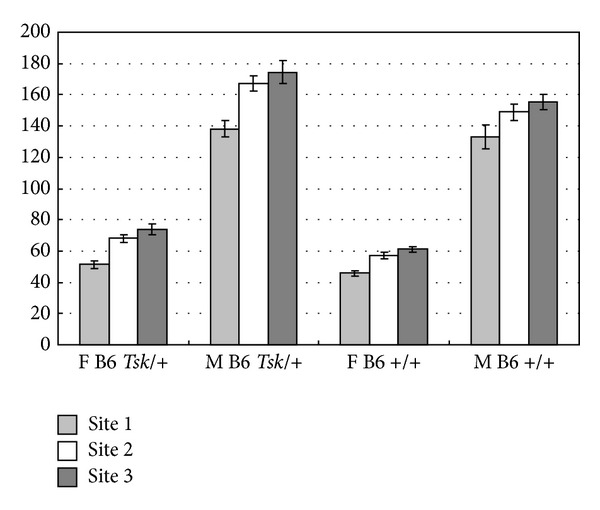
Hydroxyproline content of 4 mm skin punches from B6* Tsk*/+ and B6 +/+ mice. Site 1: proximal (light grey bar); Site 2: middle (white bar); Site 3: distal dorsum (dark grey bar). F: females and M: males. Hydroxyproline values are expressed as *μ*g/4 mm skin punch. The standard error of the mean is shown.

The results of hydroxyproline analyses for skin samples showed a striking pattern of increasing collagen content from the anterior dorsum to the posterior dorsum, with the lowest values observed from Site 1 (interscapular region) and the highest values observed from Site 3 (posterior region); this relationship was true for all groups (Table 1). Confirming the quantitation of hydroxyproline, histopathological examination of skin samples showed a progressive increase in the thickness of the dermis from the anterior dorsum to the posterior dorsum (Figure 1). Previous studies had shown significant differences in hydroxyproline content of skin samples between *Tsk*/+ mice and +/+ mice without regard to gender [6, 15, 16, 19, 20]. Although comparison of hydroxyproline content between B6 *Tsk*/+ females and B6 +/+ females showed that *Tsk*/+ females had higher hydroxyproline levels, the difference was found not to be significant for Site 1 (*P* = 0.50), highly significant for Site 2 (*P* = 0.04), and borderline significant for Site 3 (*P* = 0.12). A similar comparison of hydroxyproline content between B6 *Tsk*/+ males and B6 +/+ males showed that *Tsk*/+ males had higher hydroxyproline levels although the difference was found not to be significant for Site 1 (*P* = 0.58), highly significant for Site 2 (*P* = 0.03), and borderline significant for Site 3 (*P* = 0.08). 

 Overall comparisons showed striking differences between male and female mice. Figure 1 provides a graphical representation of these differences. *Tsk*/+ males showed 2-3-fold more hydroxyproline than their *Tsk*/+ female littermates in skin samples of the dorsum at Sites 1, 2, and 3; these differences were highly significant (*P* < 0.001). B6 +/+ males showed 2-3-fold more hydroxyproline than their B6 +/+ female littermates, and these differences were highly significant as well (*P* < 0.001). 

**Figure 2:**
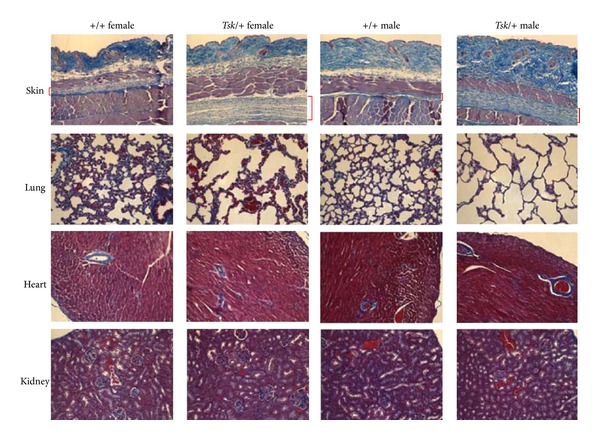
Histopathology of tissues from wild type +/+ and mutant *Tsk*/+ mice. The genotype and gender of the mice are shown at the top. The tissue is shown at the left of the figure. Skin, lung, heart, and kidney samples from mice were removed, fixed, paraffin-embedded, sectioned, and stained with Masson's trichrome. Collagen staining appears in blue. Compare the thickness of the collagen-containing dermal layer and the hypodermal or superficial fascia layer (shown in red brackets) beneath the panniculus carnosus in the skin. The age of the mice ranged between 23 and 27 weeks. Magnification is 200x.

The quantitative differences found here were confirmed by histopathological studies of skin samples (Figure 2). The thicker dermis of *Tsk*/+ males and females was evident compared to their +/+ littermates. In addition, the hypodermal layer was much thicker in both *Tsk*/+ males and females compared to their +/+ littermates (Figure 2). These results are consistent with observations by other investigators which noted the increased thickness of the hypodermal layer in *Tsk*/+ mice [21, 27, 28].

### 3.2. Hydroxyproline Content in the Lungs of C57BL/6J +/+ and *Tsk*/+ Mice

Histopathological analyses of lung tissues showed the expected enlarged alveolar spaces and fragmented thinner interstitial lung tissue in *Tsk*/+ mice compared to +/+ mice (Figure 2); this emphysema-like condition was present in both *Tsk*/+ females and males but was not observed in +/+ females and males (Figure 2). However, quantitation of hydroxyproline levels in whole lung samples did not reveal significant differences between *Tsk*/+ mice and +/+ mice (Table 2). *Tsk*/+ females had slightly lower hydroxyproline levels than +/+ females, whereas *Tsk*/+ males had slightly higher hydroxyproline levels than +/+ males. However, comparisons between the genders showed that males had much higher levels than females in the *Tsk*/+ group (*P* = 0.003) and in the +/+ group (*P* = 0.003).

### 3.3. Hydroxyproline Content in Heart Samples of C57BL/6J +/+ and *Tsk*/+ Mice

 Histopathological analysis of sections of heart tissue did not show obvious differences between *Tsk*/+ and +/+ mice (Figure 2). However, the hydroxyproline content of whole heart tissue (Table 2) in *Tsk*/+ females was increased by 8.3% on average compared to +/+ females, but this difference was not significant (*P* = 0.36). In contrast, hydroxyproline content of whole heart tissue (Table 2) in *Tsk*/+ males was increased by 20.1% on average compared to +/+ males, and this difference was significant (*P* = 0.03). These data show for the first time the specificity of the effects of the *Tsk*/+ mutation on male mice. 

### 3.4. Hydroxyproline Content in Kidney Samples of C57BL/6J +/+ and *Tsk*/+

Assays of each kidney separately (Table 2) showed that, overall, the right kidney showed slightly less hydroxyproline than the left kidney within each group, but this difference was not significant (*P* > 0.15). The hydroxyproline content of the right and left kidneys was not different in *Tsk*/+ females compared to +/+ females (*P* > 0.9). The hydroxyproline content of the right and left kidneys did not show variation in *Tsk*/+ males compared to +/+ males (*P* > 0.6). However, comparisons between *Tsk*/+ female and *Tsk*/+ male mice showed that males have ~25% more hydroxyproline on average than females, regardless of wildtype or mutant genotype, and this difference is highly significant (*P* ≤ 0.001). Histopathological analysis of sections of kidney tissue did not show obvious differences between *Tsk*/+ and +/+ mice (Figure 2). 

## 4. Discussion

In this paper, we quantitated the total collagen content in skin, heart, lung, and kidney tissue samples taken from mice of the inbred strain C57BL/6J (B6) that carry wildtype (+/+) or mutant alleles (*Tsk*/+) of the *Tsk* mutation. 

Skin collagen content along the dorsum increased from the proximal to the distal site within each group (Table 1). This trend toward increasing collagen content from proximal to middle to distal dorsum was confirmed in histopathology sections of skin samples (data not shown). This increasing trend in skin collagen content is highly significant (*P* < 0.001) comparing the proximal to middle and the middle to distal regions of the dorsum. Therefore, the site of each skin sample as well as the age of the mice is important for the consistency of results. 

Furthermore, the results demonstrate that there are highly significant differences in skin hydroxyproline levels between *Tsk*/+ males and *Tsk*/+ females (*P* < 0.001). Similar differences were previously documented between B6 +/+ males and B6 +/+ females at 2 through 16 weeks of age [25]. Our results show a significant 2-3-fold higher collagen content in older (23–27 weeks of age) *Tsk*/+ and B6 +/+ control males compared to *Tsk*/+ and B6 +/+ control females, respectively (Table 1 and Figure 2). Furthermore, staining with Masson's trichrome showed a marked increase in the thickness of the dermis plus hypodermis in both male and female *Tsk*/+ mice compared to their +/+ littermates, although the differences in dermal thickness were less pronounced in females (Figure 2). 

Histological sections of the lungs of *Tsk*/+ mice showed the typical emphysematous abnormalities described previously [5, 6, 9, 29] with increased alveolar spaces and thin or fragmented alveolar walls compared to +/+ control mice, regardless of gender (Figure 2). However, the hydroxyproline content did not significantly differ between mutant *Tsk*/+ mice and wildtype +/+ mice (Table 2). As noted for the skin, significant differences in lung collagen content were found between adult males and adult females; males had ~20% higher hydroxyproline levels than their female counterparts (*P* < 0.003). This finding is consistent with our earlier study which showed remarkable differences in hydroxyproline levels between males and females at 16 weeks of age [25].

Histological sections of the kidneys of *Tsk*/+ mice did not show obvious differences compared to +/+ control mice, regardless of gender (Figure 2). Furthermore, hydroxyproline content did not significantly differ between mutant *Tsk*/+ mice and wildtype +/+ mice (Table 2). However, as was the case with lung tissues, significant differences were found between adult males and adult females; males had ~26% higher hydroxyproline levels than their female counterparts (*P* < 0.001). 

The initial observations of the *Tsk* phenotype did not reveal a thickened dermis in the skin of *Tsk*/+ mice, although the hypodermis was substantially thicker compared to +/+ mice [5]; this observation was made in the original *Tsk* mutants, which arose on the B10.D2(58N)/Sn inbred strain. However, several subsequent studies showed substantial differences.  Table 3 shows a compiled list of the results of dermal thickness and hydroxyproline content in mouse skin published previously. In 4 out of 5 studies, the dermis of *Tsk*/+ mice was approximately 2-fold thicker than the dermis of the normal or *pa/pa* controls [6, 15, 16, 20]. However, none of these 4 studies indicated the gender of the mice in each group. Furthermore, in one study where only B6 female mice were used, the dermal thickness of *Tsk*/+ mice was not significantly higher than the dermal thickness of their +/+ counterparts [21].  Table 3 also shows that in 3 out of 4 studies, the collagen or hydroxyproline content of *Tsk*/+ mice skin ranged from 1.9- to 2.5-fold higher than the collagen or hydroxyproline content of the normal, *pa*/+, or *pa*/*pa* control skin [6, 19, 20]. However, none of these 3 studies specified the gender of the mice in each group, and in one study their age was not given. In contrast, in the single study where 3-month-old B6 female mice were used, the hydroxyproline content of *Tsk*/+ mice was only ~25% higher than the hydroxyproline content of their +/+ counterparts [21]. The data here are consistent with those of Saito et al. [21], since the *Tsk*/+ mice in our study exhibit an average increase of only ~15% in hydroxyproline content compared to +/+ mice of the same gender (Table 1).

The differences in *Tsk* collagen or hydroxyproline content between our results and those of other investigators could, in part, be due to the genetic heterogeneity of Tsk/+ mice used in previous studies which has been largely removed in the present study owing to the use of genetically homogeneous mice. Alternatively, gender, age, and site of sample differences could explain the differences between our observations and those of several studies listed in Table 3. The results of this present study highlight the importance of using gender-matched mice from inbred strains which are genetically homogeneous for studies concerning collagen content. Furthermore, studies in *Tsk*/+ mice examining the role of specific cell populations or of various interventions, such as for example, employing antibodies to specific molecules or putative therapeutic agents, may yield misleading results if they fail to take into account the important differences related to age, gender, and site of tissue sample. Thus, further investigations into the *Tsk* phenotype and its modifications must take into account the effects of different strain backgrounds, the age and gender of the mice, and the site of origin of the samples studied. 

## 5. Conclusion

This paper documents important gender and tissue related differences in collagen content in normal mice and in mice harboring a large duplication mutation in the fibrillin-1 gene (*Tsk*/+ mice). These results indicate that comparative studies of collagen content between normal and *Tsk*/+ mice skin and internal organs must take into account gender differences caused by expression of the androgen receptor. 

## Figures and Tables

**Table 1:** Hydroxyproline content in skin of B6 +/+ and B6 *Tsk*/+ mice.

Group	Sex	Age in weeks	Number of mice	Site 1 Mean ± S.E	Site 2 Mean ± S.E	Site 3 Mean ± S.E
B6 +/+	F	24–27	6	45.7 ± 1.6	57.0 ± 2.1	61.1 ± 1.6
B6 *Tsk/+ *	F	23–25	6	51.1 ± 2.5	68.0 ± 2.5	73.9 ± 3.7
				*P* = 0.50	*P* = 0.043	*P* = 0.12

B6 +/+	M	24–27	6	133.0 ± 7.8	149.4 ± 5.3	155.4 ± 5.1
B6 *Tsk/+ *	M	24–27	6	138.3 ± 5.4	167.8 ± 4.8	174.3 ± 7.4
				*P* = 0.58	*P* = 0.032	*P* = 0.076

Hydroxyproline content is expressed as *µ*g/4 mm skin punch. F: females; M: males. Site 1: proximal; Site 2: middle; Site 3: distal dorsum. S.E: standard error. Statistical comparisons were performed using the *t*-test following analysis of variance (see Section 2).

**Table 2:** Hydroxyproline content in lung, heart, and kidney of B6 +/+ and B6 *Tsk*/+ mice.

Genotype	Sex	Age in weeks	Number of mice	Lung Mean ± S.E	Heart Mean ± S.E	Kidney (L) Mean ± S.E	Kidney (R) Mean ± S.E
B6 +/+	F	24–27	5	272.6 ± 10.0	106.8 ± 6.2	120.0 ± 4.9	114.4 ± 4.3
B6 *Tsk/*+	F	23–25	6	264.5 ± 17.3	115.7 ± 3.5	120.5 ± 1.9	113.2 ± 6.8
				*P* = 0.69	*P* = 0.36	*P* = 0.96	*P* = 0.90

B6 +/+	M	24–27	6	316.7 ± 9.0	106.2 ± 5.4	152.2 ± 5.5	141.5 ± 3.4
B6 *Tsk*/+	M	24–27	6	325.2 ± 20.0	127.5 ± 9.1	149.5 ± 12.6	145.8 ± 9.8
				*P* = 0.71	*P* = 0.028	*P* = 0.81	*P* = 0.64

Hydroxyproline is expressed as *μ*g/per organ content in lung, heart, and kidney. F: females; M: males. L: left; R: right. S.E: standard error. Statistical analysis was performed using *t*-test following analysis of variance.

**Table 3:** Summary of published data showing skin thickness and hydroxyproline content in *Tsk/*+ and control mice.

Strain background	Genotype	No. of mice studied	Age	Gender	Dermal thickness (mm)	Fold change in dermal thickness^b^	Fold change in collagen or hydroxyproline content^b^	Reference
B10.D2(58N)/Sn	*Normal *	5	6 months	NG^a^	1.1 ± 0.1			Jimenez et al., 1984 [6]
B10.D2(58N)/Sn	*Tsk *	5	6 months	NG	2.2 ± 0.1	2.0	2.5	

NZB	*pa/+ *	2	NG	NG				Bocchieri et al., 1993 [19]
NZB	*Tsk/+ *	2	NG	NG			2.3	

C57BL/6	*pa/pa *	5	2-3 months	NG	~0.180			Ong et al., 1999 [15]
BALB/c, C57BL/6	*Tsk/+ IL4+/+*, *STAT6+/+ *	5	2-3 months	NG	~0.380	2.1		
BALB/c, C57BL/6	*Tsk/+ IL4−/−*, *STAT6+/+ *	5	2-3 months	NG	~0.190	1.0		
BALB/c, C57BL/6	*Tsk/+ IL4+/−, STAT6+/+ *	5	2-3 months	NG	~0.290	1.6		
BALB/c, C57BL/6	*Tsk/+ IL4+/+, STAT6+/− *	5	2-3 months	NG	~0.160	0.8		
BALB/c, C57BL/6	*Tsk/+ IL4+/+, STAT6−/− *	5	2-3 months	NG	~0.170	0.9		

C57BL/6	*pa/pa *	6	NG	NG	0.132 ± 0.042			McGaha et al., 2001 [20]
C57BL/6	*Tsk/+ pa/+ *	13	NG	NG	0.228 ± 0.072	1.7	2.0	
BALB/c, C57BL/6	*Tsk/+, Il4R*α*+/− *	9	NG	NG	0.243 ± 0.088	1.8	1.5	
BALB/c, C57BL/6	*Tsk/+, Il4R*α*−/− *	9	NG	NG	0.135 ± 0.031	1.0	0.9	
C57BL/6	*Tsk/+*, *Tgfb+/− *	16	NG	NG	0.164 ± 0.078	1.2	0.8	
C57BL/6	*+/+*, *Tgfb+/− *	3	NG	NG	0.120 ± 0.018	0.9	0.8	

C57BL/6	*pa/pa *	6	3 months	NG	0.118 ± 0.021			Kodera et al., 2002 [16]
C57BL/6	*Tsk/+ pa/+ *	13	3 months	NG	0.232 ± 0.089	1.9		
BALB/c, C57BL/6	*Tsk/+, Il4+/+ *	6	3 months	NG	0.327 ± 0.081	2.7		
BALB/c, C57BL/6	*Tsk/+, Il4+/− *	12	3 months	NG	0.205 ± 0.091	1.7		
BALB/c, C57BL/6	*Tsk/+, Il4−/− *	27	3 months	NG	0.143 ± 0.042	1.2		
BALB/c, C57BL/6	*Tsk/Tsk, Il4+/− *	5	3 months	NG	0.162 ± 0.023	1.4		
BALB/c, C57BL/6	*Tsk/Tsk, Il4−/− *	5	3 months	NG	0.139 ± 0.019	1.2		

C57BL/6	*+/+ *	NG	3 months	Female	~0.160			Saito et al., 2002 [21]
C57BL/6	*Tsk/+ *	NG	3 months	Female	~0.390	1.1	1.25	
C57BL/6, 129	*CD19 −/− *	NG	3 months	Female	~0.160	1.0	1.0	
C57BL/6, 129	*Tsk/+, CD19 −/− *	NG	3 months	Female	~0.320	1.0	1.1	

C57BL/6	*+/+ *	11	NG		0.206 ± 0.021			Del Prete et al., 2004 [22]
C57BL/6	*Tsk/+ *	8	NG		0.182 ± 0.089			

C57BL/6	*+/+ *	23	6 weeks	Male	~0.8			Baxter et al., 2005 [23]
C57BL/6	*Tsk/+ pa/+ *	34	6 weeks	Male	~1.5	2.0		

^a^NG: not given. ^b^Fold change is shown compared to control.
